# Efficacy and safety of brain–computer interface for stroke rehabilitation: an overview of systematic review

**DOI:** 10.3389/fnhum.2025.1525293

**Published:** 2025-03-06

**Authors:** Jiajun Liu, Yiwei Li, Dongjie Zhao, Lirong Zhong, Yan Wang, Man Hao, Jianxiong Ma

**Affiliations:** ^1^Tianjin Hospital, Tianjin University, Tianjin, China; ^2^The Second School of Clinical Medicine, Guangzhou University of Chinese Medicine, Guangzhou, China; ^3^Clinical Medical College of Acupuncture Moxibustion and Rehabilitation, Guangzhou University of Chinese Medicine, Guangzhou, China

**Keywords:** stroke, brain–computer interface, rehabilitation therapy, umbrella review, motor recovery

## Abstract

**Background:**

Stroke is a major global health challenge that significantly influences public health. In stroke rehabilitation, brain–computer interfaces (BCI) offer distinct advantages over traditional training programs, including improved motor recovery and greater neuroplasticity. Here, we provide a first re-evaluation of systematic reviews and meta-analyses to further explore the safety and clinical efficacy of BCI in stroke rehabilitation.

**Methods:**

A standardized search was conducted in major databases up to October 2024. We assessed the quality of the literature based on the following aspects: AMSTAR-2, PRISMA, publication year, study design, homogeneity, and publication bias. The data were subsequently visualized as radar plots, enabling a comprehensive and rigorous evaluation of the literature.

**Results:**

We initially identified 908 articles and, after removing duplicates, we screened titles and abstracts of 407 articles. A total of 18 studies satisfied inclusion criteria were included. The re-evaluation showed that the quality of systematic reviews and meta-analyses concerning stroke BCI training is moderate, which can provide relatively good evidence.

**Conclusion:**

It has been proven that BCI-combined treatment can improve upper limb motor function and the quality of daily life for stroke patients, especially those in the subacute phase, demonstrating good safety. However, its effects on improving speech function, lower limb motor function, and long-term outcomes require further evidence. Multicenter, long-term follow-up studies are needed to increase the reliability of the results.

**Clinical Trial Registration:**

https://www.crd.york.ac.uk/PROSPERO/view/CRD42024562114, CRD42023407720.

## 1 Introduction

Stroke also referred to as a cerebrovascular accident (CVA), encompasses a group of conditions caused by the sudden blockage or rupture of cerebral blood vessels, resulting in brain tissue damage (Johnson et al., 2016). As a major global public health issue, stroke is marked by high rates of incidence, prevalence, disability, recurrence, and mortality (Feigin et al., 2022; Feigin and Owolabi, 2023). In China alone, approximately 17 million individuals are currently affected by stroke, with this number projected to reach 20 million by 2024 (Longde et al., 2022). Among these patients, 80% experience varying degrees of motor, cognitive, or speech dysfunction, substantially impacting their quality of life (Stinear et al., 2020).

Effective rehabilitation for stroke patients requires early, sustained, and continuous therapeutic intervention. It is well known that motivation plays a significant role in determining the success of rehabilitation (Maclean et al., 2000; Maclean et al., 2002). Yet, conventional rehabilitation methods without variations may be repetitive and monotonous to stroke patients (Dash et al., 2019; Gorsic et al., 2017), diminishing patients’ motivation over time. Besides, conventional therapy approaches exhibit limited recovery for stroke patients with severe hemiplegia (Ramos-Murguialday et al., 2013). In contrast, BCI may provide a useful rehabilitation approach for stroke patients with severe impairment (Chaudhary et al., 2016). It offers a dynamic alternative by providing a closed-loop system with real-time monitoring, interactive elements, and sensory feedback, enabling direct interaction between the brain and computer systems. BCI technology thus facilitates intention-driven, active rehabilitation, positioning it as an ideal approach for stroke patients (Biasiucci et al., 2018; Chaudhary et al., 2016; Ganzer et al., 2020). BCI-based rehabilitation for stroke-induced aphasia may present various challenges (Kleih and Botrel, 2024), but it holds the potential to assist patients in controlling prosthetic devices, thereby aiding in the relearning of essential daily activities, such as grasping objects (Li et al., 2021; Peng et al., 2022; Wan et al., 2022; Xie et al., 2022; Zheng et al., 2021). BCI-based rehabilitation, when combined with functional or neuromuscular electrical stimulation, has demonstrated notable improvements in upper extremity function and the induction of plasticity within targeted neural pathways (Nojima et al., 2022; Peng et al., 2022). The integration of BCI with motor imagery (MI) rehabilitation training further enhances its potential, yielding significant gains in motor function, particularly for subacute stroke patients with severe impairments (Mansour et al., 2022). These features offer considerable benefits for stroke patients with functional disorders.

In the past decade, BCI-based rehabilitation has rapidly evolved and found increasing applications in clinical settings, accompanied by a surge in related research publications reflecting increased international interest (Liu et al., 2023). Numerous systematic reviews and meta-analyses have been conducted in response, though they vary widely in quality and scope. Clinical evidence supports the substantial efficacy of BCI training in stroke rehabilitation, but the reliability of these systematic reviews and meta-analyses—essential for guiding clinical practice—largely depends on their methodological quality. To date, a comprehensive re-evaluation of systematic reviews and meta-analyses focusing on BCI in stroke rehabilitation has yet to be undertaken.

Thus, this study seeks to examine the unique characteristics of BCI interventions, underscoring both their strengths and limitations in stroke rehabilitation and identifying underlying mechanisms. Utilizing ASTMR-2, PRISMA, and radar plot methodologies, this umbrella review will conduct a qualitative and quantitative assessment of the existing literature. The intended outcome is to establish a research agenda for future studies to bolster the rigor, depth, and breadth of the evidence base, validating BCI as a promising and effective rehabilitation tool for stroke patients among diverse therapeutic approaches.

## 2 Methods

### 2.1 Literature retrieval

**Figure 1:**
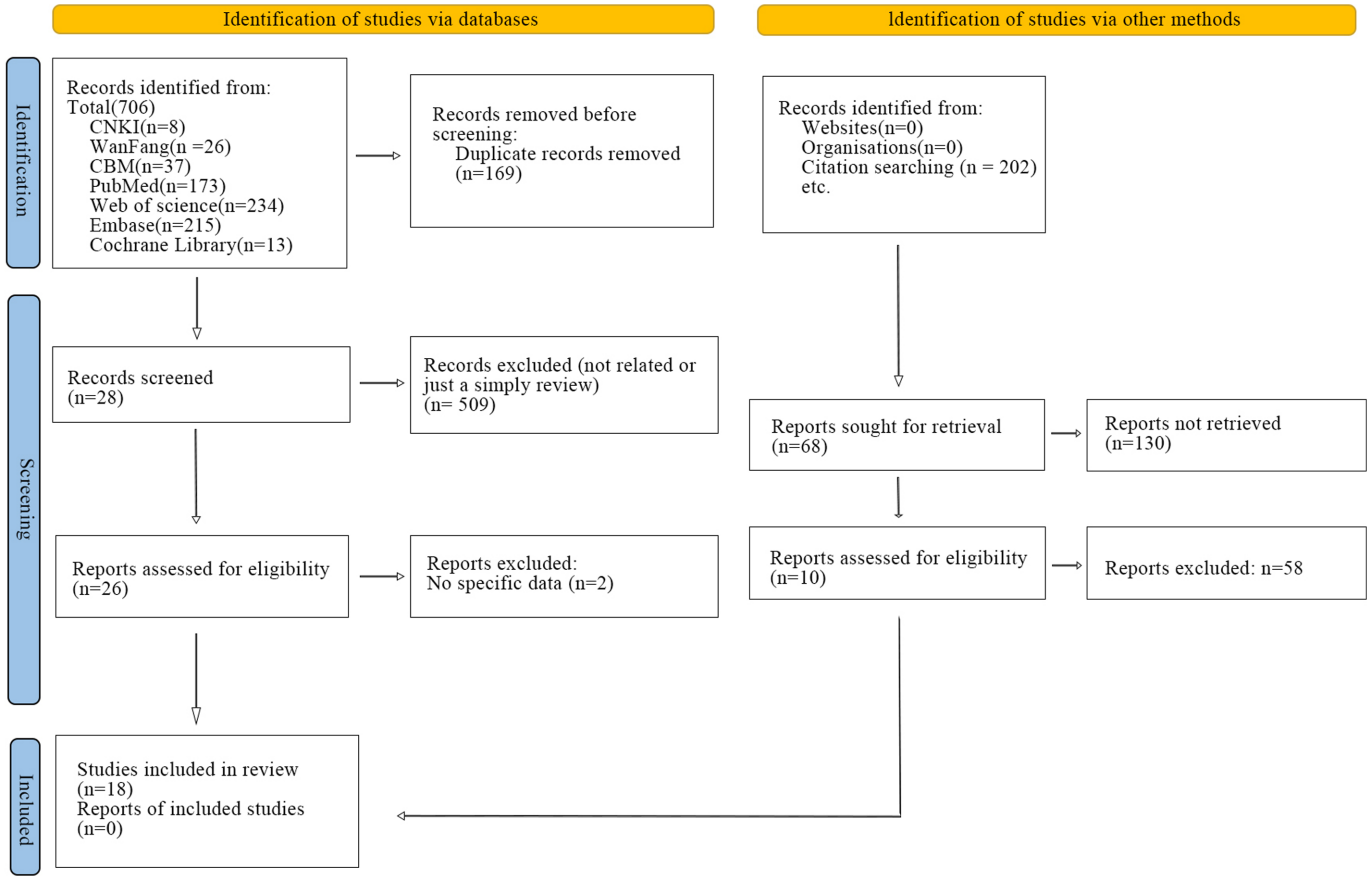
Flowchart of the study selection process.

A systematic search strategy was conducted in China National Knowledge Infrastructure (CNKI), Wan fang Data Knowledge Service Platform and Chinese Biomedical Literature Database (CBM), The Cochrane Library, PubMed, Embase, and Web of Science from the start of the database to October 14th, 2024. Additionally, forward citation tracking was identified by manually searching the included studies. The search strategy is presented in Supplementary material and a flow chart has been generated (Figure 1). This study has been registered on the international systematic evaluation registration platform PROSPERO with the registration number CRD42023407720.

### 2.2 Inclusion and exclusion criteria

The inclusion criteria were: (a) Population: stroke patients with any course of disease, gender, age, or race; (b) Interventions: BCI or BCI combined with other relative therapies; (c) Control group: other active treatment measures; (e) Outcome measurement: the primary outcome indicators focus on motor function or the ability of daily life, which can be assessed using relevant scales, such as Fugal-Mayer Assessment (FMA), the Action Research Arm Test (ARAT), Modified Barthel Index (MBI). The secondary outcome indicators target brain function, with no restrictions on the choice of assessment tools; (e) Study design: systematic reviews or meta-analysis.

The exclusion criteria were as follows: (a) not published in English or Chinese; (b) Randomized controlled trial (RCT) or clinical trials; (c) repeated publications; (d) proposal for systematic reviews or meta-analysis; (e) methodological studies; (f) abstract or translation of systematic reviews or meta-analysis.

### 2.3 Data extraction

Duplicates were first eliminated and two researchers (YWL and DJZ) independently reviewed the titles and abstracts of all articles to evaluate if they were eligible for inclusion in this umbrella review. Researchers used the same inclusion and exclusion criteria by reviewing titles and abstracts, and eligible articles were screened in full for final inclusion. Discrepancies were resolved through discussion or consultation with a third investigator (JJL).

The following information was extracted from each study: (1) publication information (authors, year of publication); (2) participant characteristics (age and sample size); (3) intervention information (type of BCI-based intervention, intervention in the control group); (4) outcome measures (type of scales, such as FMA, MBI); (5) conclusion (findings of this systematic review).

### 2.4 Assessment of methodological quality

To evaluate the methodological quality of each review, we employed AMSTAR-2 (A Measurement Tool to Assess Systematic Reviews) (Shea et al., 2017) and PRISMA (The Preferred Reporting Items for Systematic Reviews and Meta-Analyses) (Page et al., 2021). AMSTAR-2 assesses quality across 16 criteria, rated as follows: “Yes” (criterion met, score 1), “No” (criterion unmet, score 0), or “Partially Met/Not Applicable” (score 0.5). PRISMA applies a checklist with two scoring options: “Yes” (criterion met, score 1) and “No” (criterion unmet, score 0). In cases of rating discrepancies, consensus was achieved through discussion.

In addition to AMSTAR-2 and PRISMA, we evaluated methodological quality across four supplementary dimensions: publication year, study design, homogeneity, and publication bias. Each review was scored in these six areas, with specific criteria applied to each. For example, more recent publication years received higher scores. Most recent publications were to account for the latest advancements in the field and the potential relevance of newer data. Study designs were scored with RCTs and observational studies at 18 points, while other controlled trials received 14. For homogeneity, studies with *p* ≥ 0.01 and I^2^ ≤ 50% were scored at 18 points, indicating high homogeneity. In assessing publication bias, studies lacking both forest and funnel plots were classified as having a high risk of bias.

This comprehensive approach provides a robust framework for assessing the quality of systematic reviews and meta-analyses, enhancing reliability for evidence synthesis in clinical practice.

## 3 Results

### 3.1 Process and outcomes of literature screening

A total of 706 relevant studies were retrieved, including 71 from Chinese databases and 635 from English databases. Specifically, 37 from the Chinese Biomedical Literature Database, 26 from the Wan fang Database, 8 from the CNKI, 234 from Web of Science, 215 from Embase, 173 from PubMed, and 13 from The Cochrane Library. No studies were identified in other databases. After removing 169 duplicates, the titles and abstracts of the remaining studies were screened using EndNote reference management software, resulting in the exclusion of 509 articles. Ultimately, 26 studies were identified as potentially eligible, and their full texts were downloaded for a secondary review. Of these, 2 articles were further excluded as they lacked specific data. In addition, 202 studies were identified through citation searching. After 33 studies without retrieved data were removed, we identified 130 reports as potentially eligible. Of these, 58 articles were further excluded. Some systematic reviews were excluded due to their lack of quality or relevance to the study (Camargo-Vargas et al., 2021; Cha and Hwang, 2022; Monge-Pereira et al., 2017; Shou et al., 2023). Therefore, 18 studies were included in the final analysis. The detailed study selection process is illustrated in Figure 1.

### 3.2 Characteristics of the included studies

A total of 18 articles were ultimately included in the analysis, all of which have been published in domestic or international journals. The publication years ranged from 2018 to 2024. Of the included studies, two were titled “A Systematic Review” (Baniqued et al., 2021; Fu et al., 2023), seven were titled “A Systematic Review and Meta-analysis” (Bai et al., 2020; Kruse et al., 2020; Li et al., 2021; Lima et al., 2024; Mansour et al., 2022; Peng et al., 2022; Qu et al., 2022; Zhang et al., 2024), and eight were titled “Meta-analysis” (Bai et al., 2020; Cervera et al., 2018; Nojima et al., 2022; Wan et al., 2022; Xie et al., 2022; Xue et al., 2023; Yang et al., 2022; Zheng et al., 2021). Most studies examined BCI training in comparison to sham BCI or conventional treatment. Notably, one study contrasted BCI training combined with robotic assistance with standard BCI training in healthy participants (Baniqued et al., 2021). Additionally, a study reviewed BCI training against sham BCI, conventional therapy, or robotic training (Qu et al., 2022). In a further investigation, BCI training combined with transcranial direct current stimulation (tDCS) was assessed against a sham group, highlighting an exploration into adjunctive stimulation techniques (Lima et al., 2024).

In the risk of bias assessment for RCT, 13 studies employed the Cochrane Handbook for Systematic Reviews of Interventions recommended tool for assessing bias risk (Cervera et al., 2018; Kruse et al., 2020; Li et al., 2021; Lima et al., 2024; Nojima et al., 2022; Peng et al., 2022; Qu et al., 2022; Wan et al., 2022; Xie et al., 2022; Xue et al., 2023; Yang et al., 2022; Zhang et al., 2024; Zheng et al., 2021), while four used the PEDro scale (Bai et al., 2020; Baniqued et al., 2021; Fu et al., 2023; Mansour et al., 2022). One study did not employ any scale for bias risk assessment (Carvalho et al., 2019).

In the outcome measures, all included studies reported motor function outcomes, one study reported brain function (Kruse et al., 2020), and 6 studies reported ability of daily living (Li et al., 2021; Peng et al., 2022; Wan et al., 2022; Xue et al., 2023; Zhang et al., 2024; Zheng et al., 2021). The key characteristics of the included studies are presented in Table 1.

**Table 1:** Characteristics of the included studies.

Reference	Design	Age (years)^a^	Time after stroke (months)^a, b^	Studies Number (Sample Subjects) ^c^	Treatment group	Control group	Risk of bias	Outcomes	Conclusion
Cervera et al. (2018)	RCT	49.3 ~ 67.1	2.0 ~ 4.5	9(235)	BCI	Without BCI	Cochrane ROB	①: FMA, ARAT, MAS, MAL, GAS, Ashworth, MRC, ROM; ③: NIHSS, KVIQ-10; ④: MBI	Effects of BCI-based neurorehabilitation on upper-limb motor function show a medium to large effect size and can improve FMA-UE scores more than other conventional therapies.
Carvalho et al. (2019)	RCT	49.3 ~ 67.1	1.7 ~ 71.0	9 (233)	BCI combined with CR	Without Restrictions	None	①: FMA, ARAT, MAS, MAL, GAS, 3ROM, MFT; ③: VAS; ④: MBI	Neurofeedback training with BCI systems seems to promote clinical and neurophysiologic changes in stroke patients, in particular those with long-term efficacy.
Kruse et al. (2020)	RCT, Controlled Trials	40.9 ~ 64.1	Subacute/ Chronic	14 (362)	BCI	CR	Cochrane ROB	①: FMA, ARAT, MAS, MFT, MAL, MRC, GAS, 10MWT, ROM, TUG, BBS; ②: EMG, RS-fMRI, Brain symmetry index, Functional connectivity change,. Attention index; ③: NIHSS, VAS; ④: MBI	BCI training added to CR may enhance motor functioning of the upper extremity and brain function recovery in patients after a stroke.
Bai et al. (2020)	Single Group Study, Controlled Trials	40.9 ~ 67.1	2.0 ~ 73.6	33 (497)	BCI	Sham BCI or CR	PEDro scale	①: FMA, ARAT, MRC, MAL, MAS, 9-HPT, GAS, GS, BBT, JHFT, ROM; ②: EEG, EMG, NIRS, fMRI; ③: NIHSS, SIAS, ESS; ④: MBI	The use of BCI has significant immediate effects on the improvement of hemiparetic upper extremity function in patients after stroke, but the limited number of studies does not support its long-term effects.
Li et al. (2021)	RCT	41.6 ~ 72.4	0.6 ~ 39.8	14 (504)	BCI combined with CR	CR	Cochrane ROB	①: FMA-UE, FMA-LE, ARAT, WMFT; ④: MBI	BCI can improve the upper limb motor function and activities of daily life of stroke patients, and the BCI with the electrical stimulation has the best effect.
Zheng et al. (2021)	RCT	41.7 ~ 72.4	N/A	12 (347)	BCI	CR	Cochrane ROB	①: FMA, ARAT, MAL, MRC, MAS, MMT; ④: MBI	BCI training can improve limb motor function, movement, muscle strength, and activities of daily life in stroke patients, but the effect on spasticity is not obvious.
Nojima et al. (2022)	RCT, Pilot Study	40.9 ~ 66.3	2.0 ~ 4.5	16 (382)	BCI involving Neurofeedback training	Without BCI	Cochrane ROB	①: FMA, ARAT, MAL, MRC, MAS, ROM, BBT, **9-**HPT, JHFT; ②: EEG, EMG, RS-fMRI; ③: NIHSS, SIAS, ESS	This meta-analysis suggested that BCI-based training was superior to CR for motor recovery of the upper limbs in patients with stroke.
Baniqued et al. (2021)	RCT, Observational Studies (Case Report, Case–Control Study, Case Series)	44.8 ~ 62.0	2.0 ~ 7.0	30 (207)	BCI combined with a Robotic device (e.g., exoskeleton)	BCI on healthy participants	PEDro scale	①: FMA, ARAT, GS, BBT; ②: EEG, EMG	They identified large heterogeneity in reporting emphasizing the need to develop a standard protocol for assessing technical and clinical outcomes so that the necessary evidence based on efficiency and efficacy can be developed.
Yang et al. (2022)	RCT	41.6 ~ 66.3	Subacute/ Chronic	13 (258)	BCI	Sham BCI or CR	Cochrane ROB	①: FMA-UE, MFT, ARAT, MAL, MRC, MAS, ROM, GAS, WMFT; ④: MBI	BCI training was shown to be effective in promoting upper limb motor function recovery in post-stroke patients.
Wan et al. (2022)	RCT	41.8 ~ 64.1	0.6 ~ 15.4	13 (470)	BCI	CR	Cochrane ROB	①: FMA, MAS; ④: MBI	BCI has a significant improvement effect on the upper limb motor function and the ADL of stroke patients, and the statistical results are stable.
Peng et al. (2022)	RCT	41.6 ~ 72.4	0.6 ~ 66.0	16 (488)	BCI or BCI combined with CR	CR or Blank Control or Other Treatment without BCI	Cochrane ROB	①: FMA, MAS; ④:MBI.	BCI therapy or BCI combined with other therapies such as conventional rehabilitation training and motor imagery training can improve upper limb dysfunction after stroke and enhance the quality of daily life.
Qu et al. (2022)	Single Group Study, Controlled Trials	40.9 ~ 62.9	2.0 ~ 66.0	19 (413)	BCI	Sham BCI or CR or Robot Training without BCI	Cochrane ROB	①: FMA, ARAT, MAS, WMFT, GAS, MAL ②: EEG, EMG, task-fMRI, fMRI	The use of BCI-robot systems has significant improvement on the motor function recovery of the hemiparetic upper limb, and there is a sustaining effect.
Mansour et al. (2022)	RCT	N/A	Subacute/ Chronic	12 (298)	BCI	Without Restrictions	PEDro scale	①: FMA-UE, MRC, MAS, MAL, ARAT, GAS, WMFT; ③: ESS; ④: MBI	Future BCI-based stroke rehabilitation studies could use “intention of movement of the impaired hand” as the BCI mental practice, the band power features as the BCI classification features, and the functional electrical stimulation as the BCI feedback
Xie et al. (2022)	RCT	41.6 ~ 66.3	2.1 ~ 39.8	17 (410)	BCI	Sham BCI or CR without BCI	Cochrane ROB	①: FMA, MRC, MAS, ARAT, GAS, MAL, ROM, KVIQ-10, WMFT; ③: NIHSS, ESS; ④: MBI	BCI-based training improved upper limb motor function and ADL in post-stroke patients.
Xue et al. (2023)	RCT, Observational Studies (Case–Control Study, Case Series)	46.6 ~ 72.43	2.7 ~ 67.6	9 (226)	BCI	Without Restrictions	Cochrane ROB	①: FMA-UE, MAS; ④: MBI	BCI training could effectively improve the recovery effect of upper limb function in stroke patients.
Fu et al. (2023)	RCT	N/A	N/A	15 (373)	BCI	CR or Sham BCI	PEDro scale	①: FMA, ARAT, JHFT, MRC, MAS, GAS, MAL; ②: EEG; ④: MBI	To optimize BCI rehabilitation training, we should focus on patients ‘difficulties during BCI training to help them complete grasp motions, finger extension, thumb opposition, and other complex motions.
Lima et al. (2024)	RCT, Parallel or Crossover Studies	52.2 ~ 63.9	Subacute/ Chronic	9 (262)	tDCS combined with BCI	Sham tDCS with BCI or only BCI	Cochrane ROB	Motor function and Brain functional connectivity	There is no evidence of the effect of tDCS associated with BCI in post-stroke recovery. tDCS is of no additional benefit over BCI alone.
Zhang et al. (2024)	RCT	NA	NA	25 (730)	BCI	Sham BCI or CR without BCI	Cochrane ROB	①: FMA-UE, ARAT, MAL, MAS; ④: MBI	BCI has favorable long-term outcomes. In terms of total duration of training, < 12 h of training may lead to better rehabilitation, and ≥ 12 h of training did not show an advantage over the control group.

^aData is reported as mean ranges of the experimental group. bIf the article does not mention the mean value, it is presented as stage types of stroke. CSample subjects of the experimental group. The meaning of each number representative: ① Motor function; ② Brain function; ③ Clinical outcomes; ④ Ability of daily life; ARAT, the Action Research Arm Test; ADL, ability of daily life; BBS: Berg Balance Scale; BBT: Box and Block Test; CR, conventional rehabilitation; tDCS, transcranial direct current stimulation; EEG, electroencephalography; EMG, electromyography; ESS: European Stroke Scale; FMA, Fugl-Mayer assessment; FMA-UE, Fugl-Mayer assessment for upper extremity; FMA-LE, Fugl-Mayer assessment for lower extremity; GAS, goal attainment score; GS: Grip strength; 9-HPT: Nine-Hole Peg Test; JHFT: Jebsen Hand Function Test; KVIQ-10: Kinesthetic and Visual Imagery Questionnaire-10; NIHSS: National Institute Health Stroke Scale; NIRS, near-infrared spectroscopy; MAS, Modified Ashworth Scale; MAL, Motor Activity Log; MBI, Modified Barthel Index; MFT, Modified Function Test; MRC, Medical Research Council scale for muscle strength; MMT, Manual Muscle Test; 10MWT, 10 m walking test; PEDro, physiotherapy evidence-based database; RCT, random controlled trials; ROB, risk-of-bias tool; ROM, range of motion; RS-fMRI, resting-state functional magnetic resonance imaging; SIAS, stroke impairment assessment set; TUG, timed up and go Test; VAS, visual analogue scale; WMFT, Wolf Motor Function Test.^

### 3.3 Comparison of publication years

The clinical relevance of each study is directly influenced by the year of publication, the scope of coverage, and the temporal span of the research. This article includes 18 systematic reviews and meta-analyses, with the rank of the 2023 and 2024 publications assigned as 18. Among the included systematic reviews and meta-analyses, the earliest was published in 2018 (*n* = 1), while the most recent appeared in 2024 (*n* = 1). Additional studies include two from 2023, seven from 2022, four from 2021, two from 2020, and one each from 2019 and 2018.

### 3.4 AMSTAR-2 evaluation

The AMSTAR-2 methodological quality assessment comprises 16 criteria. Among the 18 reviewed studies, the scores ranged from 6 to 13. Specifically, one study scored 6, one scored 6.5, one scored 7, one scored 9, two scored 10, two scored 11, two scored 12, four studies scored 12.5, and four achieved a score of 13, reflecting a generally moderate overall quality. When evaluating the AMSTAR-2 results based on seven key domains related to the validity of findings (items 2, 4, 7, 9, 11, 13, and 15), the studies were classified into four categories: high, moderate, low, and critically low. Twelve studies were rated as critically low (Bai et al., 2020; Baniqued et al., 2021; Carvalho et al., 2019; Cervera et al., 2018; Fu et al., 2023; Mansour et al., 2022; Peng et al., 2022; Qu et al., 2022; Wan et al., 2022; Xie et al., 2022; Yang et al., 2022; Zheng et al., 2021), six as low (Kruse et al., 2020; Li et al., 2021; Lima et al., 2024; Nojima et al., 2022; Xue et al., 2023; Zhang et al., 2024).

Key issues included: (1) Regarding item 2 (whether the review protocol was established before conducting the systematic review, and whether discrepancies between the protocol and study were explained), only three studies were rated as “yes,” (Kruse et al., 2020; Nojima et al., 2022; Zhang et al., 2024) while five were rated as “partially yes,” (Baniqued et al., 2021; Fu et al., 2023; Lima et al., 2024; Xue et al., 2023) and the rest were rated as “no.” (2) For item 7 (whether the authors provided a list of excluded studies with reasons for exclusion), only two studies were rated as “yes” (Carvalho et al., 2019; Xue et al., 2023), while all others were rated as “no.” Detailed evaluation results are provided in Supplementary materials.

### 3.5 PRISMA evaluation

The PRISMA checklist, with a total score of 27 points, was used to evaluate 18 articles, with scores ranging from 13 to 27. Several factors contributed to the lower scores: ① Only six articles provided a registration number (Baniqued et al., 2021; Fu et al., 2023; Kruse et al., 2020; Li et al., 2021; Nojima et al., 2022; Qu et al., 2022), while the remaining 12 did not; ② Except for five studies (Cervera et al., 2018; Fu et al., 2023; Kruse et al., 2020; Lima et al., 2024; Yang et al., 2022), the abstracts in most of the included articles were insufficiently detailed.

### 3.6 Radar plots

**Figure 2:**
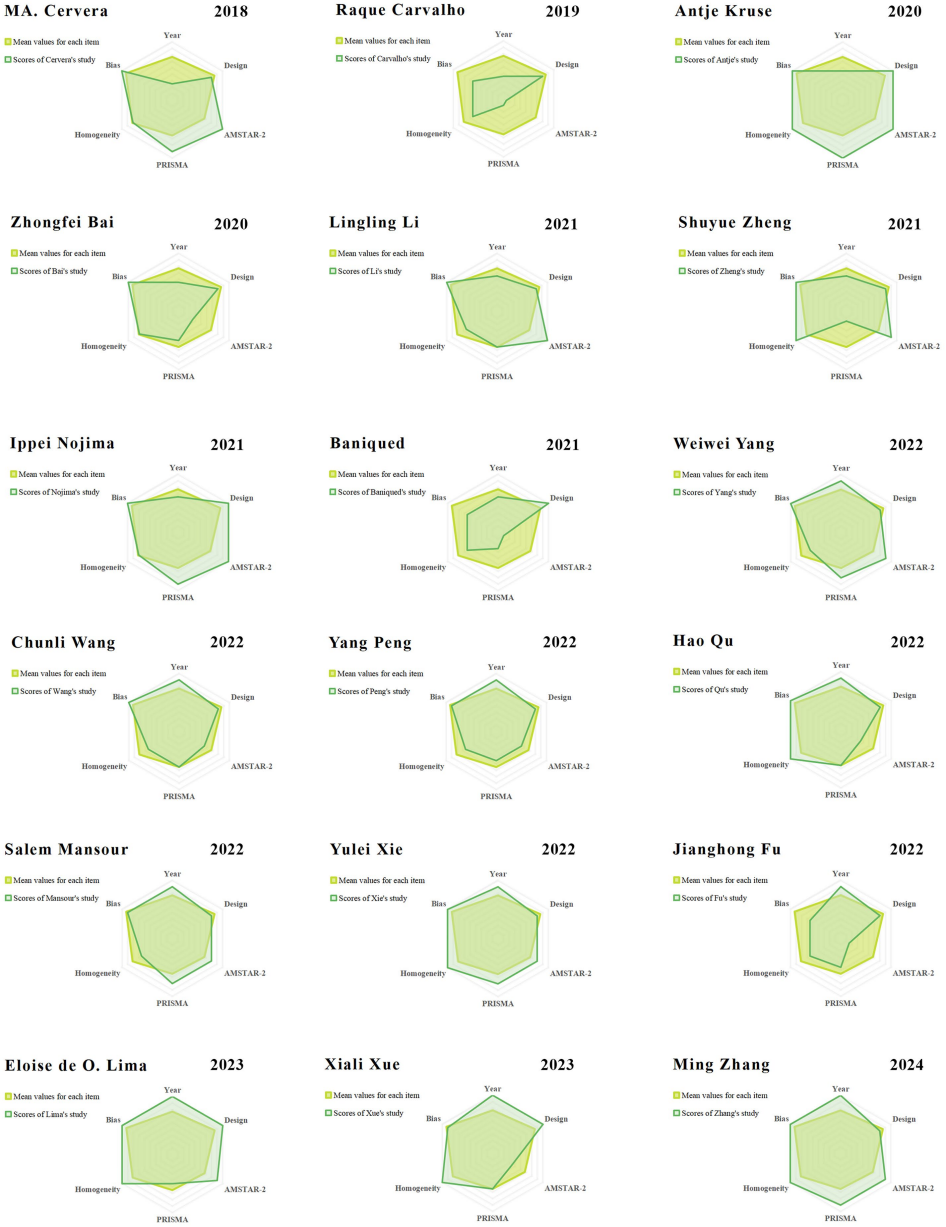
The radar plots of the included studies.

A comprehensive analysis was conducted based on the visual representation of the radar chart and the mean ranks of the various studies. This investigation identified 11 studies with relatively high quality (Cervera et al., 2018; Kruse et al., 2020; Li et al., 2021; Lima et al., 2024; Mansour et al., 2022; Nojima et al., 2022; Qu et al., 2022; Xie et al., 2022; Xue et al., 2023; Yang et al., 2022; Zhang et al., 2024), each exhibiting balanced and reliable scores across the evaluation criteria, while the remaining seven studies exhibited relatively moderate quality (Bai et al., 2020; Baniqued et al., 2021; Carvalho et al., 2019; Fu et al., 2023; Peng et al., 2022; Wan et al., 2022; Zheng et al., 2021). Notably, the majority of these articles received relatively moderate methodological quality. For additional details, refer to Figure 2, and the multi-dimensional evaluation criteria are outlined in Table 2.

**Table 2:** Scoring of the six dimensions of the included studies.

References	Year	Design	AMSTAR-2	PRISMA	Homogeneity	Publication Bias	Mean scores
Cervera et al. (2018)	2018 (5)	RCT (14)	13 (18)	25 (16)	Low (14)	Forest plot, Funnel plot (18)	14.17
Carvalho et al. (2019)	2019 (7)	RCT (14)	6 (1)	13 (2)	None (11)	None (11)	7.67
Kruse et al. (2020)	2020 (9)	RCT, Controlled Trials (18)	13 (18)	27 (18)	High (18)	Forest plot, Funnel plot (18)	16.50
Bai et al. (2020)	2020 (9)	Single Group Study, Controlled Trials (14)	9 (5)	22 (9)	Low (14)	Forest plot, Funnel plot (18)	11.50
Li et al. (2021)	2021 (11)	RCT (14)	13 (18)	23 (11)	None (11)	Forest plot, Funnel plot (18)	13.83
Zheng et al. (2021)	2021 (11)	RCT (14)	12.5 (16)	19 (3)	High (18)	Forest plot, Funnel plot (18)	13.33
Nojima et al. (2022)	2021 (11)	RCT, Pilot Study (18)	13 (18)	25 (16)	Low (14)	Forest plot, Funnel plot (18)	15.83
Baniqued et al. (2021)	2021 (11)	RCT, Observational Studies (Case Report, Case–Control Study, Case Series) (18)	6.5 (2)	20 (5)	None (11)	None (11)	9.67
Yang et al. (2022)	2022 (16)	RCT (14)	12.5 (16)	24 (14)	None (11)	Forest plot, Funnel plot (18)	14.83
Wan et al. (2022)	2022 (16)	RCT (14)	11 (9)	23 (11)	None (11)	Forest plot, Funnel plot (18)	13.17
Peng et al. (2022)	2022 (16)	RCT (14)	11 (9)	22 (9)	None (11)	Forest plot (16)	12.50
Qu et al. (2022)	2022 (16)	Single Group Study, Controlled Trials (14)	10 (7)	23 (11)	High (18)	Forest plot, Funnel plot (18)	14.00
Mansour et al. (2022)	2022 (16)	RCT (14)	12 (14)	24 (14)	None (11)	Forest plot (16)	14.17
Xie et al. (2022)	2022 (16)	RCT (14)	12 (14)	24 (14)	High (18)	Forest plot, Funnel plot (18)	15.67
Xue et al. (2023)	2023 (18)	RCT, Observational Studies (Case–Control Study, Case Series) (18)	10 (7)	23 (11)	High (18)	Forest plot (16)	14.67
Fu et al. (2023)	2022 (16)	RCT (14)	7 (3)	22 (9)	None (11)	None (11)	10.67
Lima et al. (2024)	2023 (18)	RCT, Parallel or Crossover Studies (18)	12.5 (16)	22 (9)	High (18)	Forest plot, Funnel plot (18)	16.17
Zhang et al. (2024)	2024 (18)	RCT (14)	12.5 (16)	25 (16)	High (18)	Forest plot, Funnel plot (18)	16.67
Mean scores	13.33	15.11	11.50	11.00	14.22	16.50	13.51

Scoring criteria of publication year: More recent publications achieve higher scores; Scoring criteria of study design: Studies including both RCT and observational studies scored 18, while those with only RCT or controlled trials scored 14; AMSTAR and PRISMA scores according to the original scores (from highest to lowest): 18, 16, 14, 11, 9, 7, 5, 3, 2, 1; Scoring criteria of homogeneity: If *P* ≥ 0.01 and *I*^2^ ≤ 50%, it was high homogeneity scored 18; Scoring criteria of publication bias: If the study does not consist of forest plot and funnel plot, it was defined as high publication bias.

## 4 Discussion

### 4.1 Summary of the evidence

Currently, this article encompasses a total of 18 studies, comprising 4 Chinese publications and 14 English publications, all published between 2018 and 2023. The research designs include randomized controlled trials and observational studies, or a combination of both. Among these, the study by Zhang et al. (2024) attained the highest quality, with a score of 16.67 while the study by Raque Carvalho exhibited the lowest quality (Carvalho et al., 2019), with a score of 7.67. The AMSTAR-2 scores for the included studies ranged from 6 to 13, while the PRISMA scores ranged from 12 to 27. The majority of the studies exhibited a moderate degree of heterogeneity, with only 6 studies demonstrating significantly low homogeneity (Baniqued et al., 2021; Carvalho et al., 2019; Fu et al., 2023). Most studies utilized forest plot analyses, with only 3 studies not employing this analytical method (Baniqued et al., 2021; Carvalho et al., 2019; Fu et al., 2023). The overall mean rank score of the included literature was merely 13.04.

Systematic reviews and meta-analyses provide a structured approach for evaluating result reliability. With an increase in reviews and analyses on stroke rehabilitation via BCI, methodological inadequacies are increasingly apparent, often due to insufficient adherence to methodological standards and issues in study design. These observations underscore the importance of rigor in systematic reviews, signaling the need to re-evaluate literature quality to establish a robust, evidence-based foundation for rehabilitation applications.

The primary reason for the low quality of these studies is their inadequate methodological and reporting standards. Key areas requiring improvement include: (1) conducting systematic and comprehensive searches, with attention to publication language and inclusion of gray literature to reduce publication bias; (2) providing thorough documentation of studies, covering subjects, intervention methods, outcomes, study types, settings, and funding sources; (3) applying validated tools to assess the risk of bias in included studies, with funnel plots as a standard for detecting potential publication bias; (4) explicitly disclosing funding sources and other supporting contributions.

### 4.2 Efficacy of BCI for post-stroke rehabilitation

Stroke is associated with a high rate of disability, with 70–80% of stroke patients experiencing sequelae such as cognitive impairment (Collaborators, 2023), motor dysfunction, and speech deficits, which can lead to severe long-term disability (Hosoi et al., 2022; Liu et al., 2021; Rangaraju et al., 2015). Among the functional impairments resulting from stroke, motor dysfunction—particularly weakness in the upper and lower extremities—not only severely hampers a patient’s ability to grasp and walk but also significantly impacts their quality of life and their access to participate in social activities. Consequently, the restoration of upper and lower limb motor function has become a central focus of stroke rehabilitation (Mane et al., 2020).

The effects of BCI-based neurorehabilitation on upper-limb motor function have been well-documented, with BCI training leading to greater improvements in FMA-UE scores compared to conventional therapies. This finding has been confirmed by 12 systematic reviews and meta-analyses (Bai et al., 2020; Cervera et al., 2018; Kruse et al., 2020; Li et al., 2021; Mansour et al., 2022; Nojima et al., 2022; Peng et al., 2022; Wan et al., 2022; Xie et al., 2022; Xue et al., 2023; Yang et al., 2022; Zheng et al., 2021). Furthermore, multiple randomized controlled trials (RCTs) have consistently confirmed the effectiveness of BCI on upper-limb motor function. Overall, BCI therapy, particularly when combined with other interventions like conventional rehabilitation and motor imagery training, has the potential to significantly enhance motor function recovery, though the specific aspects of upper-limb motor function that are improved, as well as the occurrence of related adverse events such as headaches and nausea, require further investigation and attention (Kruse et al., 2020).

Apart from motor function, BCI therapy, particularly when combined with other interventions such as conventional rehabilitation training and motor imagery training, can enhance the quality of daily life (Li et al., 2021; Peng et al., 2022; Wan et al., 2022; Xie et al., 2022). Action observation training plus BCI-FES (Functional Electronic Stimulation) group demonstrated significant improvement in the MBI compared to the control group, suggesting enhanced motor function and daily activities in stroke patients (Lee et al., 2022).

In addition to its benefits for motor function and daily life, BCI applications are showing potential for improving speech abilities in stroke patients, though the research in this area is still in its early stages. A BCI-based language training approach is feasible and effective in improving language skills in 10 stroke patients with aphasia, inducing sustained recovery and enhancing brain plasticity without affecting non-linguistic skills (Musso et al., 2022). Another study assessed visual P300 BCI for chronic post-stroke aphasia rehabilitation, finding improvements in speech and quality of life, but the results are preliminary due to a small sample and missing data (Kleih and Botrel, 2024).

Besides, the use of BCI-robot systems has significantly improved motor function recovery of the hemiparetic upper limb, and there is a sustaining effect. However, the meta-analysis showed no statistical difference between the experimental group (BCI-robot) and the control group (Qu et al., 2022; Qu et al., 2022), further research is needed to confirm this. Moreover, there is no evidence of the effect of tDCS associated with BCI in post-stroke recovery (Lima et al., 2024).

Regarding the disease course, six studies consistently concluded that BCI therapy was more effective in the subacute phase (Cervera et al., 2018; Kruse et al., 2020; Peng et al., 2022; Qu et al., 2022; Xue et al., 2023; Yang et al., 2022), with only one review offering a contradictory perspective that BCI training appears to be equally effective in both subacute (<6 months) and chronic (>6 months) stroke patients (Nojima et al., 2022), with no statistically significant differences observed in treatment outcomes based on the duration since stroke onset. This suggests that BCI may be more effective in the recovery of subacute stroke patients. Since improvements are possible in the chronic phase, they will be slower (Zhao et al., 2022).

Whether the BCI training has long-term efficacy is still controversial. Neurofeedback training with BCI seems to promote clinical and neurophysiologic changes in stroke patients, in particular those with long-term efficacy (Carvalho et al., 2019). However, a systematic review and meta-analysis concluded that there is currently insufficient evidence to support the long-term efficacy of BCI (Bai et al., 2020). The latest meta-analysis included more studies to further explore this issue and concluded that BCI has favorable long-term outcomes (Zhang et al., 2024).

In summary, BCI-based rehabilitation has shown promising results in enhancing motor function recovery, particularly in the upper limbs and improving daily life quality for stroke patients. While its effectiveness in the subacute phase is more pronounced, further research is needed to confirm its long-term efficacy and potential benefits in other areas such as speech abilities.

### 4.3 Mechanism of BCI for post stroke rehabilitation

Post-stroke rehabilitation training may strengthen connections between neurons in existing neural pathways and lead to the formation of new neural connections (Wieloch and Nikolich, 2006). The recovery of brain function is closely linked to motor function recovery. Studies have detected a positive correlation between the increase in the laterality index value and the scoring in the FMA scale in the BCI group (Pichiorri et al., 2015; Ramos-Murguialday et al., 2013). Enhancing the excitability of the ipsilateral motor cortex is considered crucial for the recovery of motor function in hemiparetic upper limbs. There was a higher power of desynchronization over the ipsilesional central area during MI tasks than pre-intervention, indicating greater activation of the ipsilesional motor system after BCI training. This suggests that the underlying mechanism of BCI involves the activation of ipsilateral motor neurons, leading to stronger desynchronization in the ipsilateral hemisphere (McDonnell and Stinear, 2017). Therefore, the neural mechanism of BCI underlying the clinical effects is very likely to be relevant to the ipsilesional activation in the primary and secondary motor cortices (Li et al., 2014; Mihara et al., 2013). Besides, changes in the integrity (fractional anisotropy value) of the corticospinal tract of the regions of interest were positively correlated with changes in motor function (Halder et al., 2013), which indicated that it may be another factor in the improvement in motor function.

There are three main types of BCI tasks most commonly used for stroke rehabilitation: motor imagery (MI) based, intention movement (IM) based, and action observation-based BCI. In motor imagery (MI)-based BCI, patients imagine moving the impaired hand without actual movement, whereas in the intention of movement (IM)-based BCI, patients try to move the impaired hand physically, if possible. Intention movement-based BCI is also known as movement attempt-based BCI or motor attempt-based BCI (Chowdhury et al., 2018; Fu et al., 2023; Mansour et al., 2022). Both the MI-based and IM-based BCI have previously been widely investigated and differential neural mechanisms have been proposed: MI-related network and Hebbian plasticity (Ang et al., 2014; Biasiucci et al., 2018). BCI enhances neural circuit activation through IM rather than mere MI, increasing patient engagement and attention, which may contribute to its efficacy. The effect size for motor function recovery was higher in studies using IM compared to those using MI, though the difference between the two subgroups was not statistically significant (Chaudhary et al., 2016; Chowdhury et al., 2018).

Most popular BCI systems used in stroke rehabilitation are based on non-invasive EEG, and feedback is usually visual (Mihara et al., 2013). Realistic feedback, such as a virtual hand movement, might be preferable to more abstract feedback, given the possibility that its observation may itself lead to an activation of mirror neurons in the sensorimotor areas (Lucca, 2009; Pfurtscheller et al., 2008). When the patient attempts to move their paralyzed limb using motor imagery, they receive visual cues (e.g., a virtual limb moving on a screen) that reflect their brain signals. This reinforces the brain’s motor circuits, helping to strengthen neural connections related to the imagined movement (Brunner et al., 2024). Studies should be performed to verify if realistic visual feedback combined with robotic feedback in stroke patients can improve upper limb function even more (Carvalho et al., 2019).

### 4.4 Strengths and limitation

To the best of our knowledge, no previous studies have conducted a re-evaluation of systematic reviews and meta-analyses on this topic. This represents the first such assessment. Our umbrella review was meticulously conducted in accordance with the AMSTAR-2 and PRISMA guidelines.

The inclusion of literature was restricted to only two languages (Chinese and English), with no search conducted for printed or gray literature, potentially leading to the omission of relevant studies. A total of only 18 articles were included, with an average quality ranking score of 13.04, indicating that the limited number and overall moderate quality of the included studies may reasonably support our findings. The evaluation of literature quality and the assessment of evidence quality may have been influenced by the subjective biases of the reviewers, leading to the possibility of reporting bias.

### 4.5 Implications for future research

In terms of functional recovery, most current studies have focused on upper limb rehabilitation. Future research should further explore the effects of BCI on lower limb recovery. Additionally, beyond motor function, there aren’t any commonly used and proven non-invasive BCI rehabilitation techniques that address post-stroke aphasia particularly, presenting both challenges and opportunities in this area. Multicenter, long-term follow-up studies could provide more robust evidence on the long-term efficacy of BCI for motor function recovery in stroke patients. Moreover, the optimal duration of treatment also requires further investigation.

## 5 Conclusion

In summary, the re-evaluation indicates that the systematic reviews and meta-analyses concerning BCI training for stroke patients is relatively in good quality, which could provide a rigorous evidence. Nevertheless, future meta-analysts still need to enhance the methodological rigor and reporting quality of their studies, adhering strictly to the standards outlined by the AMSTAR-2 and PRISMA guidelines in their analytical discussions, thereby providing higher-quality evidence for clinical practice.

BCI-combined treatment has shown great potential in effectively improving upper limb function in stroke patients, particularly those in the subacute phase. It also demonstrates good safety and enhances the quality of daily life. However, further evidence is required to confirm its impact on enhancing speech function, lower limb motor function, and long-term outcomes. Future research should focus on these areas to comprehensively evaluate the potential of BCI-based interventions in stroke rehabilitation.

## Data Availability

The datasets presented in this study can be found in online repositories. The names of the repository/repositories and accession number(s) can be found in the article/Supplementary material.
