# Supplementary material for: Efficacy and safety of brain–computer interface for stroke rehabilitation: an overview of systematic review
Source: Front Hum Neurosci. 2025 Mar 6;19:1525293. doi: 10.3389/fnhum.2025.1525293 (PMC11922947; doi:10.3389/fnhum.2025.1525293)
Supplement: Supplementary file 1 [file Data_Sheet_1.DOCX]

Supplementary Material

[1 Search strategy 1](#_Toc22279)

[2 Supplementary Tables 4](#_Toc8510)

# Search strategy

**Pubmed**

1. "Stroke"[Mesh] OR "Strokes" OR "Cerebrovascular Accident" OR "Cerebrovascular Accidents" OR "CVA(Cerebrovascular Accident)" OR "CVAs(Cerebrovascular Accident)" OR "Cerebrovascular Apoplexy" OR "Apoplexy, Cerebrovascular" OR "Vascular Accident, Brain" OR "Brain Vascular Accident" OR "Brain Vascular Accidents" OR "Vascular Accidents, Brain" OR "Cerebrovascular Stroke" OR "Cerebrovascular Strokes" OR "Stroke, Cerebrovascular" OR "Strokes, Cerebrovascular" OR "Apoplexy" OR "Cerebral Stroke" OR "Cerebral Strokes" OR "Stroke, Cerebral" OR "Strokes, Cerebral" OR "Stroke, Acute" OR "Acute Stroke" OR "Acute Strokes" OR "Strokes, Acute" OR "Cerebrovascular Accident, Acute" OR "Acute Cerebrovascular Accident" OR "Acute Cerebrovascular Accidents" OR "Cerebrovascular Accidents, Acute"
2. "Brain-Computer Interfaces"[Mesh] OR "Brain Computer Interfaces" OR "Interface, Brain-Computer" OR "Interfaces, Brain-Computer" OR "Brain-Computer Interface" OR "Brain Computer Interface" OR "Brain-Machine Interfaces" OR "Brain-Machine Interface" OR "Interface, Brain-Machine" OR "Interfaces, Brain-Machine" OR "Brain Machine Interface" OR "Brain Machine Interfaces" OR "Interface, Brain Machine" OR "Interfaces, Brain Machine" OR "Machine Interface, Brain" OR "Machine Interfaces, Brain"
3. "meta-analysis"[Publication Type] OR "meta-analysis as topic"[MeSH Terms] OR "meta-analysis"[All Fields]
4. "systematic review"[Publication Type] OR "systematic reviews as topic"[MeSH Terms] OR "systematic reviews"[All Fields]
5. review[All Fields]
6. #3 OR #4 OR #5
7. #1 AND #2 AND #6

**Embase**

1. 'cerebrovascular accident'/exp OR 'accident, cerebrovascular' OR 'acute cerebrovascular lesion' OR 'acute focal cerebral vasculopathy' OR 'acute stroke' OR 'apoplectic stroke' OR 'apoplexia' OR 'apoplexy' OR 'blood flow disturbance, brain' OR 'brain accident' OR 'brain attack' OR 'brain blood flow disturbance' OR 'brain insult' OR 'brain insultus' OR 'brain vascular accident' OR 'cerebral apoplexia' OR 'cerebral insult' OR 'cerebral stroke' OR 'cerebral vascular accident' OR 'cerebral vascular insufficiency' OR 'cerebro vascular accident' OR 'cerebrovascular arrest' OR 'cerebrovascular failure' OR 'cerebrovascular injury' OR 'cerebrovascular insufficiency' OR 'cerebrovascular insult' OR 'cerebrum vascular accident' OR 'cryptogenic stroke' OR 'CVA' OR 'insultus cerebralis' OR 'ischaemic seizure' OR 'ischemic seizure' OR 'stroke' OR 'thrombotic stroke' OR 'cerebrovascular accident'
2. 'brain computer interface'/exp OR 'bci system (brain-computer interface)' OR 'bci-controlled neuroprosthetic' OR 'brain computer interface system' OR 'brain computer interfaces' OR 'brain computing interface' OR 'brain machine interface' OR 'brain-computer interface' OR 'brain-computer interfaces' OR 'brain-computer interfacing system' OR 'brain-machine interface' OR 'cerebellum-machine interface' OR 'cerebral-computer interfaces' OR 'direct neural interface' OR 'mind-machine interface' OR 'brain computer interface'
3. 'systematic review'/exp OR 'review, systematic' OR 'systematic review' OR 'meta analysis'/exp OR 'analysis, meta' OR 'meta-analysis' OR 'meta analysis' OR 'meta analysis' OR review
4. #1 AND #2 AND #3

**Cochrane Library**

1. Stroke OR "Cerebral Strokes" OR "Cerebral Stroke" OR "Vascular Accident, Brain" OR "Strokes" OR "Apoplexy, Cerebrovascular" OR "Apoplexy" OR "Cerebrovascular Apoplexy" OR "Cerebrovascular Stroke" OR "Stroke, Cerebrovascular" OR "Cerebrovascular Accident" OR "Brain Vascular Accident" OR "Cerebrovascular Accidents" OR "Brain Vascular Accidents" OR "Cerebrovascular Strokes" OR "Stroke, Cerebral" OR "CVAs (Cerebrovascular Accident)" OR "Strokes, Cerebrovascular" OR "CVA (Cerebrovascular Accident)" OR "Vascular Accidents, Brain" OR "Strokes, Cerebral" OR "Cerebrovascular Accidents, Acute" OR "Cerebrovascular Accident, Acute" OR "Acute Strokes" OR "Strokes, Acute" OR "Acute Stroke" OR "Acute Cerebrovascular Accident" OR "Acute Cerebrovascular Accidents" OR "Stroke, Acute"
2. "Brain-Computer Interfaces" OR "Interfaces, Brain Machine" OR "Brain Machine Interfaces" OR "Interface, Brain-Machine" OR "Machine Interfaces, Brain" OR "Interfaces, Brain-Machine" OR "Brain-Machine Interfaces" OR "Machine Interface, Brain" OR "Interface, Brain Machine" OR "Brain Machine Interface" OR "Brain-Machine Interface" OR "Brain Computer Interfaces" OR "Interfaces, Brain-Computer" OR "Interface, Brain-Computer" OR "Brain-Computer Interface" OR "Brain Computer Interface"
3. #1 AND #2 in Cochrane Reviews

**Web of Science**

1. TS=("Stroke" OR "Strokes" OR "Cerebrovascular Accident" OR "Cerebrovascular Accidents" OR "CVA(Cerebrovascular Accident)" OR "CVAs(Cerebrovascular Accident)" OR "Cerebrovascular Apoplexy" OR "Apoplexy, Cerebrovascular" OR "Vascular Accident, Brain" OR "Brain Vascular Accident" OR "Brain Vascular Accidents" OR "Vascular Accidents, Brain" OR "Cerebrovascular Stroke" OR "Cerebrovascular Strokes" OR "Stroke, Cerebrovascular" OR "Strokes, Cerebrovascular" OR "Apoplexy" OR "Cerebral Stroke" OR "Cerebral Strokes" OR "Stroke, Cerebral" OR "Strokes, Cerebral" OR "Stroke, Acute" OR "Acute Stroke" OR "Acute Strokes" OR "Strokes, Acute" OR "Cerebrovascular Accident, Acute" OR "Acute Cerebrovascular Accident" OR "Acute Cerebrovascular Accidents" OR "Cerebrovascular Accidents, Acute")
2. TS=("Brain-Computer Interfaces" OR "Brain Computer Interfaces" OR "Interface, Brain-Computer" OR "Interfaces, Brain-Computer" OR "Brain-Computer Interface" OR "Brain Computer Interface" OR "Brain-Machine Interfaces" OR "Brain-Machine Interface" OR "Interface, Brain-Machine" OR "Interfaces, Brain-Machine" OR "Brain Machine Interface" OR "Brain Machine Interfaces" OR "Interface, Brain Machine" OR "Interfaces, Brain Machine" OR "Machine Interface, Brain" OR "Machine Interfaces, Brain")
3. TS=("meta-analysis" OR "meta-analysis as topic" OR "meta-analysis" OR "systematic review" OR "systematic reviews as topic" OR "systematic reviews" OR review)
4. #1 AND #2 AND #3

**WangFang Database**

1. "Brain Computer Interface" or "Brain Machine Interface" or "Brain-computer Interaction"
2. "Stroke" or "hemiplegia" or " cerebrovascular accident" or "cerebral infarction"
3. "Systematic Reviews" or "reviews" or "Meta analysis"
4. #1 AND #2 AND #3

**China National Knowledge Infrastructure (CNKI, Chinese Database)**

1. SU=('Brain Computer Interface' + 'Brain Machine Interface' + 'Brain-computer Interaction')
2. SU=('Stroke' + 'hemiplegia' + ' cerebrovascular accident' + 'cerebral infarction')
3. SU=('Systematic Reviews' + 'reviews' + 'Meta analysis')
4. #1 AND #2 AND #3

**Chinese Biomedical Literature Database(CBM, Chinese Database)**

1. ("Brain Computer Interface" OR "Brain Machine Interface" OR "Brain-computer Interaction")
2. ("Stroke" OR "hemiplegia" OR " cerebrovascular accident" OR "cerebral infarction")
3. ("Systematic Reviews" OR "reviews" OR "Meta analysis")
4. #1 AND #2 AND #3
5. **Supplementary Tables**

Supplementary Tables 1. Scores of PRISMA

| Study ID  Items | 1 | 2 | 3 | 4 | 5 | 6 | 7 | 8 | 9 | 10 | 11 | 12 | 13 | 14 | 15 | 16 | 17 | 18 |
| --- | --- | --- | --- | --- | --- | --- | --- | --- | --- | --- | --- | --- | --- | --- | --- | --- | --- | --- |
| Title | 2 | 1 | 1 | 1 | 1 | 1 | 1 | 1 | 1 | 1 | 1 | 1 | 1 | 1 | 1 | 1 | 1 | 1 |
| Abstract | 1 | 0 | 1 | 0 | 0 | 0 | 0 | 1 | 0 | 0 | 0 | 0 | 0 | 0 | 0 | 1 | 1 | 0 |
| Background | 0 | 1 | 1 | 1 | 1 | 1 | 1 | 1 | 1 | 1 | 1 | 1 | 1 | 1 | 1 | 1 | 1 | 1 |
| Purpose | 1 | 0 | 1 | 1 | 0 | 0 | 0 | 1 | 1 | 0 | 0 | 1 | 0 | 1 | 1 | 1 | 1 | 1 |
| Regester | 1 | 0 | 1 | 0 | 1 | 0 | 1 | 0 | 0 | 1 | 0 | 0 | 0 | 0 | 0 | 0 | 0 | 1 |
| Inclusion | 0 | 0 | 1 | 1 | 1 | 1 | 1 | 0 | 1 | 1 | 1 | 1 | 1 | 1 | 0 | 1 | 1 | 1 |
| Database | 1 | 1 | 1 | 1 | 1 | 1 | 1 | 1 | 1 | 1 | 1 | 1 | 1 | 1 | 1 | 1 | 1 | 1 |
| Search | 1 | 1 | 1 | 0 | 1 | 0 | 0 | 1 | 1 | 1 | 1 | 1 | 1 | 1 | 1 | 1 | 1 | 0 |
| Selection | 1 | 1 | 1 | 1 | 1 | 1 | 1 | 1 | 1 | 1 | 1 | 1 | 1 | 1 | 1 | 1 | 1 | 1 |
| Data extraction | 1 | 0 | 1 | 1 | 1 | 1 | 1 | 1 | 1 | 1 | 1 | 1 | 1 | 1 | 1 | 1 | 1 | 1 |
| Data entry | 1 | 1 | 1 | 1 | 1 | 1 | 1 | 1 | 1 | 1 | 1 | 1 | 1 | 1 | 1 | 1 | 1 | 1 |
| Bias in individual studies | 1 | 0 | 1 | 1 | 1 | 1 | 1 | 1 | 1 | 1 | 1 | 1 | 1 | 1 | 1 | 0 | 0 | 1 |
| Summary effect indicators | 1 | 0 | 1 | 1 | 1 | 1 | 1 | 1 | 1 | 1 | 1 | 1 | 1 | 1 | 1 | 1 | 1 | 1 |
| Comprehensive analysis method | 1 | 1 | 1 | 1 | 1 | 1 | 0 | 1 | 1 | 1 | 1 | 1 | 1 | 1 | 1 | 1 | 1 | 1 |
| Research bias | 1 | 0 | 1 | 1 | 1 | 1 | 1 | 1 | 0 | 1 | 1 | 1 | 1 | 0 | 1 | 0 | 0 | 1 |
| Other analysis | 1 | 1 | 1 | 1 | 1 | 1 | 0 | 1 | 0 | 1 | 1 | 0 | 1 | 1 | 1 | 1 | 1 | 1 |
| Research selection | 1 | 1 | 1 | 1 | 1 | 1 | 1 | 1 | 1 | 1 | 1 | 1 | 1 | 1 | 1 | 1 | 1 | 1 |
| Research Features | 1 | 1 | 1 | 1 | 1 | 1 | 1 | 1 | 1 | 1 | 1 | 1 | 1 | 1 | 1 | 1 | 1 | 1 |
| Study internal bias | 1 | 0 | 1 | 0 | 1 | 0 | 0 | 1 | 1 | 1 | 1 | 1 | 1 | 1 | 1 | 0 | 0 | 1 |
| The results of single study | 1 | 0 | 1 | 1 | 1 | 1 | 1 | 1 | 1 | 1 | 1 | 1 | 1 | 1 | 1 | 1 | 1 | 1 |
| Synthesis of Results | 1 | 0 | 1 | 1 | 1 | 1 | 1 | 1 | 1 | 1 | 1 | 1 | 1 | 1 | 1 | 1 | 1 | 1 |
| Bias between studies | 1 | 0 | 1 | 1 | 1 | 1 | 1 | 0 | 1 | 1 | 0 | 1 | 1 | 1 | 0 | 0 | 0 | 1 |
| Additional analysis | 1 | 1 | 1 | 1 | 1 | 0 | 1 | 1 | 1 | 1 | 1 | 1 | 1 | 1 | 1 | 1 | 1 | 1 |
| Summary of evidence | 1 | 1 | 1 | 1 | 0 | 0 | 0 | 1 | 1 | 1 | 1 | 1 | 1 | 1 | 1 | 1 | 1 | 1 |
| Limitation | 1 | 0 | 1 | 1 | 1 | 1 | 1 | 1 | 1 | 1 | 1 | 0 | 1 | 1 | 1 | 1 | 1 | 1 |
| Conclusion | 1 | 1 | 1 | 1 | 1 | 1 | 1 | 1 | 1 | 1 | 1 | 1 | 1 | 1 | 1 | 1 | 1 | 1 |
| Funding | 1 | 0 | 1 | 0 | 0 | 0 | 1 | 1 | 1 | 1 | 0 | 1 | 1 | 1 | 1 | 1 | 1 | 1 |
| Total scores | 25 | 13 | 27 | 22 | 23 | 19 | 20 | 24 | 23 | 25 | 22 | 23 | 24 | 24 | 23 | 22 | 22 | 25 |

***Study ID:** ^1^Cervera 2018, ^2^Carvallho 2019, ^3^Antje Kruse 2020, ^4^Zhongfei Bai 2020, ^5^Lingling Li 2021, ^6^Shuyue Zheng 2021, ^7^Bandiqued 2021, ^8^Weiwei Yang 2022, ^9^Chunli Wang 2022, ^10^Nojima 2021,^11^Yang Peng 2022, ^12^Qu 2022, ^13^Salem Mansour 2022, ^14^Yulei Xie 2022, ^15^Xiali Xue 2023, ^16^Jianghong Fu 2022, ^17^Lima 2023, ^18^Ming Zhang 2023.

Supplementary Tables 2. Scores of ASTMAR-2

| Study ID  Items | 1 | 2 | **3** | 4 | **5** | 6 | 7 | 8 | 9 | **10** | 11 | 12 | 13 | 14 | **15** | 16 | **17** | **18** |
| --- | --- | --- | --- | --- | --- | --- | --- | --- | --- | --- | --- | --- | --- | --- | --- | --- | --- | --- |
| 1.Did the research questions and inclusion criteria for the review include the components of PICO? | 1 | 0 | 1 | 1 | 1 | 1 | 1 | 1 | 1 | 1 | 1 | 1 | 1 | 1 | 1 | 1 | 1 | 1 |
| 2.Did the report of the review contain an explicit statement that the review methods were established prior to the conduct of the review and did the report justify any significant deviations from the protocol? | 0 | 0 | 1 | 0 | 0.5 | 0 | 0.5 | 0 | 0 | 1 | 0 | 0 | 0 | 0 | 0.5 | 0.5 | 0.5 | 1 |
| 3.Did the review authors explain their selection of the study designs for inclusion in the review? | 1 | 1 | 1 | 0 | 1 | 1 | 0 | 1 | 1 | 1 | 1 | 0 | 1 | 1 | 0 | 0 | 1 | 1 |
| 4.Did the review authors use a comprehensive literature search strategy? | 0.5 | 0.5 | 0.5 | 0.5 | 0.5 | 0.5 | 0.5 | 0.5 | 0.5 | 0.5 | 0.5 | 0.5 | 0.5 | 0.5 | 1 | 1 | 0.5 | 0.5 |
| 5.Did the review authors perform study selection in duplicate? | 1 | 1 | 1 | 1 | 1 | 1 | 1 | 1 | 1 | 1 | 1 | 1 | 1 | 1 | 1 | 0 | 1 | 1 |
| 6.Did the review authors perform data extraction in duplicate? | 1 | 1 | 1 | 1 | 1 | 1 | 1 | 1 | 0 | 1 | 1 | 1 | 1 | 1 | 1 | 0 | 1 | 1 |
| 7.Did the review authors provide a list of excluded studies and justify the exclusions? | 0 | 1 | 0 | 0 | 0 | 0 | 0 | 0 | 0 | 0 | 0 | 0 | 0 | 0 | 1 | 0 | 0 | 0 |
| 8.Did the review authors describe the included studies in adequate detail? | 0.5 | 0.5 | 0.5 | 0.5 | 1 | 1 | 0.5 | 1 | 0.5 | 0.5 | 0.5 | 0.5 | 0.5 | 0.5 | 0.5 | 1 | 0.5 | 1 |
| 9.Did the review authors use a satisfactory technique for assessing the risk of bias in individual studies that were included in the review? | 1 | 0 | 1 | 0 | 1 | 1 | 0 | 1 | 1 | 1 | 1 | 1 | 1 | 1 | 1 | 0 | 1 | 1 |
| 10.Did the review authors report on the sources of funding for the studies included in the review? | 1 | 0 | 0 | 0 | 0 | 0 | 0 | 0 | 0 | 0 | 0 | 0 | 0 | 0 | 0 | 0.5 | 0 | 0 |
| 11.If meta-analysis was performed did the review authors use appropriate methods for statistical combination of results? | 1 | 0 | 1 | 1 | 1 | 1 | 0 | 1 | 1 | 1 | 1 | 1 | 1 | 1 | 0.5 | 0.5 | 1 | 1 |
| 12.If meta-analysis was performed, did the review authors assess the potential impact of RoB in individual studies on the results of the meta-analysis or other evidence synthesis? | 1 | 0 | 1 | 1 | 1 | 1 | 0 | 1 | 1 | 1 | 1 | 1 | 1 | 1 | 0.5 | 0.5 | 1 | 0 |
| 13.Did the review authors account for RoB in individual studies when interpreting/ discussing the results of the review? | 1 | 0 | 1 | 1 | 1 | 1 | 1 | 1 | 1 | 1 | 1 | 0 | 1 | 1 | 1 | 0 | 1 | 1 |
| 14.Did the review authors provide a satisfactory explanation for, and discussion of, any heterogeneity observed in the results of the review? | 1 | 0 | 1 | 1 | 1 | 1 | 0 | 1 | 1 | 1 | 1 | 1 | 1 | 1 | 0.5 | 0.5 | 1 | 1 |
| 15.If they performed quantitative synthesis did the review authors carry out an adequate investigation of publication bias (small study bias) and discuss its likely impact on the results of the review? | 1 | 0 | 1 | 0 | 1 | 1 | 0 | 1 | 1 | 1 | 0 | 1 | 1 | 1 | 0.5 | 0 | 1 | 1 |
| 16.Did the review authors report any potential sources of conflict of interest, including any funding they received for conducting the review? | 1 | 1 | 1 | 1 | 1 | 1 | 1 | 1 | 1 | 1 | 1 | 1 | 1 | 1 | 0 | 1 | 1 | 1 |
| Total score | 13 | 6 | 13 | 9 | 13 | 12.5 | 6.5 | 12.5 | 11 | 13 | 11 | 10 | 12 | 12 | 10 | 7 | 12.5 | 12.5 |
| Quality level | CL | CL | L | CL | L | CL | CL | CL | CL | L | CL | CL | CL | CL | L | CL | L | L |

***Study ID:** ^1^Cervera 2018, ^2^Carvallho 2019, ^3^Antje Kruse 2020, ^4^Zhongfei Bai 2020, ^5^Lingling Li 2021, ^6^Shuyue Zheng 2021, ^7^Bandiqued 2021, ^8^Weiwei Yang 2022, ^9^Chunli Wang 2022, ^10^Nojima 2021,^11^Yang Peng 2022, ^12^Qu 2022, ^13^Salem Mansour 2022, ^14^Yulei Xie 2022, ^15^Xiali Xue 2023, ^16^Jianghong Fu 2022, ^17^Lima 2023, ^18^Ming Zhang 2023. **Abbreviation:** C**L**: Critically Low; **L**: Low.
